# Retraction: DNA methylation-mediated repression of exosomal miR-652-5p expression promotes oesophageal squamous cell carcinoma aggressiveness by targeting PARG and VEGF pathways

**DOI:** 10.1371/journal.pgen.1011269

**Published:** 2024-05-07

**Authors:** The PLOS Genetics Editors

Following the publication of this article [1], concerns were raised regarding results presented in Figs 5, 7, and S1. Specifically,

- The Fig 6D EC109 VRGFA panel of [1] appears similar to the Fig 5C KYSE 450 FOXO3 panel of [2].
- The Fig 5F KYSE150 mimic panel of [1] appears similar to the Fig 5D TE-1 inhibitor panel of [3].
- The Fig 7E right Lenti-inhibitor panel of [1] appears similar to the Fig 7E right Lenti-inhibitor panel of [4, retracted in 5], despite the studies describing experiments using different cell lines.
- The Fig S1G NC Migration and NC Invasion panels appear similar.

This article [1] is also linked to a larger group of articles connected by concerns about image reuse.

In light of these image concerns that question the reliability and validity of the reported data, the *PLOS Genetics* Editors retract this article.

JY agreed with the retraction. PG, ML, HW, WW, and GS did not agree with the retraction. DW, SC, ZY, JZ, and YN either did not respond directly or could not be reached.
